# Comparison between N-803 and Erdafitinib in Bacillus Calmette-Guérin-Unresponsive Non-Muscle-Invasive Bladder Cancer

**DOI:** 10.3390/cancers16203445

**Published:** 2024-10-11

**Authors:** Anusha Gupta, Shiv Verma, Sanjay Gupta

**Affiliations:** 1 Department of Urology, School of Medicine, Case Western Reserve University, Cleveland, OH 44106, USA; anushagupta300@gmail.com (A.G.); sxv304@case.edu (S.V.); 2 Faculty of Science Program, University of Western Ontario, London, ON N6A 5A5, Canada; 3 The Urology Institute, University Hospitals Cleveland Medical Center, Cleveland, OH 44106, USA; 4 Department of Pharmacology, Case Western Reserve University, Cleveland, OH 44016, USA; 5 Department of Pathology, Case Western Reserve University, Cleveland, OH 44016, USA; 6 Department of Nutrition, Case Western Reserve University, Cleveland, OH 44016, USA; 7 Division of General Medical Sciences, Case Comprehensive Cancer Center, Cleveland, OH 44106, USA

## 1. Introduction

Bladder cancer, the sixth most common cancer and the tenth leading cause of cancer-related death worldwide, remains a significant global health concern [1]. In the United States, non-muscle-invasive bladder cancer (NMIBC), which affects the inner lining of the bladder, accounts for 75% of all bladder cancer cases [2]. The American Urological Association and the European Association of Urology recommend that high-risk patients undergo intravesical Bacillus Calmette-Guérin (BCG) therapy for 1–3 years [3]. BCG has been the standard treatment for NMIBC for over 40 years, leveraging both cytotoxic effects and an induced immune response to eliminate cancer cells. After urothelial cells internalize BCG, immune cells like NK cells, macrophages, monocytes, and dendritic cells are activated, initiating a robust immune response. However, despite BCG’s success since its introduction in 1976, fewer than half of NMIBC patients avoid recurrence, and many progress to muscle-invasive bladder cancer [3,4]. This progression underscores the need for less invasive, more effective treatments to prevent the high morbidity and mortality associated with radical cystectomy after BCG failure. The editorial aims to evaluate two promising treatments—N-803, recently approved by the FDA, and the targeted therapy Erdafitinib—by comparing their efficacy, side effects, costs, and mechanisms. Studies by Chamie et al. [5] on N-803 combined with BCG, and by Catto et al. [6] on Erdafitinib, serve as the basis for determining which treatment may be more suitable for high-risk BCG-unresponsive NMIBC patients. 

## 2. Background

Study 1—An open-label, multicenter study by Chamie et al. [5] assessed the efficacy of the interleukin-15 (IL-15) superagonist Nogapendekin alfa inbakicept (NAI), also known as Anktiva^®^ N-803, in combination with BCG for BCG-resistant NMIBC patients. The study aimed to determine whether patients would exhibit a significant complete response to NAI, a drug that stimulates the proliferation and activation of NK, CD8+, and CD4+ T cells [7]. Patients with carcinoma in situ (CIS), with or without Ta/T1 papillary disease, were placed into cohorts A and C, while those with confirmed Ta/T1 papillary NMIBC were assigned to cohort B. A total of 171 participants, both male and female, over 18 years old were enrolled, with most being of European descent and having a median age in their 70s. Cohorts A and B received six weekly intravesical instillations of 400 μg of NAI combined with 50 mg of BCG, while cohort C received only 400 μg of NAI. The primary endpoint for cohorts A and C was complete response (CR) at 3 or 6 months, while cohort B's primary endpoint was disease-free survival 12 months after the initial treatment. At a median follow-up of 23.9 months, cohort A showed a 71% CR rate, and cohort C showed a 20% CR rate at 3 months. Cohort B exhibited a 55.4% disease-free survival rate at a median follow-up of 19.3 months. Most patients experienced mild to moderate adverse effects (grades 1–2), supporting the tolerability and efficacy of NAI combined with BCG. This promising emerging treatment for NMIBC continues to show potential as a viable alternative to BCG therapy.

Study 2—Erdafitinib, also known as Balversa^®^, is a targeted therapy that inhibits fibroblast growth factor receptors (FGFR), which are frequently mutated in NMIBC [8]. Catto et al. [6] conducted a phase II trial involving 73 patients, randomly assigning them in a 2:1 ratio to receive either oral erdafitinib or intravesical chemotherapy. To assess and compare the efficacy of erdafitinib, BCG-resistant patients with FGFR3 gene mutations were administered either 6 mg of erdafitinib daily in 28-day cycles for up to 2 years (following a reduction from an initial 8 mg dose due to tolerance issues) or chemotherapy. The chemotherapy group received 40 mg of mitomycin C or 2000 mg of gemcitabine weekly for at least four doses, followed by monthly maintenance for 6 months. The primary endpoint was recurrence-free survival (RFS). Erdafitinib demonstrated superior outcomes, as the median RFS was not reached, while the chemotherapy group showed a median RFS of 11.6 months, highlighting the extended efficacy of erdafitinib. The hazard ratio of 0.28 further supported the drug's prolonged benefit compared to intravesical chemotherapy. However, the use of erdafitinib was associated with more severe grade 3 or more adverse events despite its greater efficacy.

## 3. Discussion

Although clinical trials are ongoing for both NAI with BCG and erdafitinib independently, there is a notable lack of direct comparative studies to determine which is the more practical alternative to BCG. Research on N-803 (NAI) and erdafitinib highlights how their distinct mechanisms of action contribute to their higher efficacy in different patient populations. NAI works by activating and rapidly increasing NK, CD8+, and CD4+ T cells, enhancing the immune system's ability to destroy cancer cells regardless of the presence of mutations [7]. In contrast, erdafitinib targets and inhibits FGFR, which can be mutated in tumor cells [8]. While erdafitinib has shown high efficacy in a smaller subset of patients with FGFR mutations, as evidenced by its inability to reach a median RFS, NAI with BCG offers a broader application by eradicating cancer cells without requiring specific molecular markers, achieving a high complete response (CR) across a wider patient population. The patient selection in both studies was limited, but the group receiving NAI with BCG was more diverse. Although most participants were older and of European descent, the NAI with BCG group was more versatile compared to the erdafitinib group, which required patients to have an FGFR mutation. This variation in patient selection highlights the broader applicability of NAI with BCG and provides more reliable results. Equally important to the treatment’s effectiveness is the safety of patients following clinical trials. Adverse events can be classified into categories ranging from mild and moderate to severe, life-threatening, and death-related, depending on the severity of the event [9]. With the overarching goal of improving patient outcomes, NAI with BCG appears more appealing, as patients in this group predominantly experienced mild to moderate (grade 1 to 2) adverse events, while those receiving erdafitinib encountered more severe (grade 3 or higher) adverse events. The reason for this difference is unclear, but erdafitinib's systemic nature may contribute to the more severe side effects. A scientific study indicates that, in the United States, the anticipated cost of N-803 (Anktiva^®^) is between USD 100,000 and 150,000 for a complete treatment course, which typically spans several months. This cost may vary based on the required number of doses and the overall treatment duration. Meanwhile, the average monthly cost of erdafitinib (Balversa^®^) is approximately USD 20,000 to 25,000, although this can differ significantly due to various factors, including geographic location, insurance coverage, and specific treatment protocols. While both treatments are costly, erdafitinib's accessibility is further limited by its reliance on molecular testing to identify patients with FGFR mutations. Molecular testing can be challenging due to the scarcity of available testing kits or the exhaustion of tissue samples [10]. The feasibility of identifying genetic alterations is low, whereas NAI can trigger an immune response to attack cancer cells without requiring such molecular markers. Both therapies are highly effective, but NAI offers potential for long-term improvements, while erdafitinib excels in achieving rapid tumor reductions.

## 4. Conclusions

The most suitable treatment alternative following BCG recurrence will ultimately depend on an individual patient's circumstances and conditions. Given the lack of direct head-to-head comparisons and the current data available for the two discussed alternatives, NAI combined with BCG seems to be a more favorable option compared to erdafitinib and intravesical chemotherapy. An ideal treatment should be defined by its balance of applicability, safety, accessibility, and efficacy, rather than merely its effectiveness for a specific patient group. Further studies are needed to validate the efficacy of NAI over erdafitinib, as well as to explore effective treatments that may be applicable to a wider range of NMIBC patients.
